# Quantum Cascade Laser Infrared Spectroscopy for Online Monitoring of Hydroxylamine Nitrate

**DOI:** 10.1155/2018/7896903

**Published:** 2018-09-23

**Authors:** Marissa E. Morales-Rodriguez, Joanna McFarlane, Michelle K. Kidder

**Affiliations:** ^1^Oak Ridge National Laboratory, USA; ^2^The Bredesen Center at the University of Tennessee Knoxville, USA

## Abstract

We describe a new approach for high sensitivity and real-time online measurements to monitor the kinetics in the processing of nuclear materials and other chemical reactions. Mid infrared (Mid-IR) quantum cascade laser (QCL) high-resolution spectroscopy was used for rapid and continuous sampling of nitrates in aqueous and organic reactive systems, using pattern recognition analysis and high sensitivity to detect and identify chemical species. In this standoff or off-set method, the collection of a sample for analysis is not required. To perform the analysis, a flow cell was used for in situ sampling of a liquid slipstream. A prototype was designed based on attenuated total reflection (ATR) coupled with the QCL beam to detect and identify chemical changes and be deployed in hostile environments, either radiological or chemical. The limit of detection (LOD) and the limit of quantification (LOQ) at 3*σ* for hydroxylamine nitrate ranged from 0.3 to 3 and from 3.5 to 10 g·L^−1^, respectively, for the nitrate system at three peaks with wavelengths between 3.8 and 9.8 *μ*m.

## 1. Introduction

The monitoring of chemical processing in hazardous or extreme conditions challenges methods that rely on sampling followed by offline analysis. Continuous processes with reactive species are particularly difficult to control and would benefit from active online monitoring of reagents or products or both. Nuclear isotope separations depend on careful control of redox chemistry, using reactive species such as hydroxylamine nitrate, HAN, to change the oxidation state of actinides dissolved in aqueous solution. Hence, we describe a spectroscopic method that could be used to monitor HAN reactions in real time. Because its flexibility, the method could be applied to any aqueous species with absorption in the mid-infrared.

Vibrational IR spectroscopy is a tool that offers the selectivity required for identifying molecular species as IR absorptions are characteristic and specific to molecular groups. Vibrational spectra can be interpreted to give thermal energies of IR-active compounds, allowing these to be included in chemical kinetic and dynamical models. Traditionally, IR transmittance is not utilized to characterize aqueous solutions because of the absorption of H_2_O, but advances in Mid-IR FTIR and the incorporation of the attenuated total reflection accessory (ATR) make it usable for aqueous solution chemistry [1–3]. The same principle of minimizing matrix absorption by using ATR was employed here. As an IR source, we used a set of four quantum cascade lasers (QCL). Potential advantages of the QCL system over a broadband source such as that used in an FTIR include portability because an evacuated light path is not required, spectral resolution based on the laser linewidth, and enhanced sensitivity through high peak power of the excitation laser [4–7]. A recent study by Pengel and colleagues [8] demonstrated the feasibility of using QCLs to monitor chemicals in solution in a static system and Alcaráz and colleagues used an external cavity QCL for measurements of proteins in the mid-IR [9]. In the work described here, the goal was to demonstrate the capability of a QCL-ATR compact and off-set system to continuously monitor (with samples taken every minute) an aqueous phase reaction in a nuclear application.

The utility of QCL standoff detection of molecules has been demonstrated in the solid and gas phase at ORNL, e.g., methane in field experiments and in the detection of explosive dust collected on solid surfaces [10–16]. However, many chemical processes occur in solution phase, and involve different molecules with distinguishing functional groups. In aqueous solution, there are two issues that need to be addressed, the high background and spectral selectivity. Hence, Raman is usually the method of choice for vibrational spectroscopy as it avoids background absorption from H_2_O. Because of selection rules, Raman is generally much less sensitive than IR absorption, unless methods such as surface enhanced Raman are used [17, 18]. For instance, Raman has been used to monitor the degradation of anion-exchange resins used for the separation of plutonium isotopes in highly acidic conditions, e.g., Buscher et al. [19]. Van Staden and colleagues cite a detection limit for both nitrate and nitrate as 500 mg/L [20]. Resonance Raman has been used to study nitrate and nitrite in wastewater treatment processes, with detection limits of 7 *μ*g [21]. This method depends on far UV excitation; however, this method becomes unfeasible for use in applications involving high concentrations of nitrate because self-absorption becomes problematic at concentrations above 3.5 mM.

The QCL-ATR system was used to monitor and assay nitrate-nitrite chemistry representative of the process for plutonium-238 production for NASA deep space missions. The chemical processing of neptunium-237 targets after irradiation involves several steps to (a) separate fission products, (b) separate the neptunium and plutonium, and (c) make purification and polishing. This process achieves separation of neptunium and plutonium through redox chemistry and selective liquid-liquid extraction from nitric acid solution (where the target is dissolved) in a tributyl phosphate (TBP)-organic mixture. Recovery of the plutonium from the organic phase needs introduction of hydroxylamine nitrate (NH_3_OH^+^·NO_3_^−^) or HAN that is used to reduce Pu(IV) to Pu(III). Hydroxylamine, or HA, is classified as a self-reactive substance [22]. The autocatalytic reaction scheme that takes place in nitric acid solution is given in Reaction (1), showing the conversion of nitric to nitrous acid [23, 24], and the decomposition of NH_3_OH^+^, Reaction (2).

(1) 2HNO3+NH2OH→3HNO2+H2O

(2) HNO2+NH3OH+→N2O+2H2O+H+

As Reactions (1) and (2) progress consuming NH_3_OH^+^, in strong nitric acid the amount of HNO_2_ can increase causing an uncontrolled reaction that can affect the recovery of the plutonium. Hence, it is important to be able to monitor the processes continuously, which is not possible with offline methods of analysis. Commonly employed methods to analyze hydroxylamine nitrate include reacting it with compounds that are spectroscopically active, such as a titration with Griess reagent, [25] or by high performance liquid chromatography [26].

Spectroscopy promises to fulfill the requirements of online analysis and selectivity for nitrate and nitrite species [27, 28]. However, there is no direct spectroscopic measurement in the UV-visible region or mid-IR for HAN in water [29]. We have developed a new method of in situ or online measurement in aqueous solution, which can be used to effectively monitor key species such as NO_3_^−^ and HNO_2_ involved in the redox process chemistry of actinides. This prototype can be incorporated into the reaction system for continuous monitoring of the reaction progress to provide rapid quantitative and qualitative analysis. The mechanisms of the prototype, data collection, and data analysis are discussed in the next section.

IR spectra of HA and derivatives have been observed in inert gas matrices and calculated by self-consistent field methods [30], observed in the gas phase as n-ethyl hydroxylamine and hydroxylamine chloride [27], and observed in molecular beams [31]. The reported spectrum of hydroxylamine chloride shows that hydroxylamine exhibits active absorption behavior with multiple peaks in the regions of ~4 and 7-12 *μ*m, coincident with the spectral region covered by the QCL system utilized in this project. Hence, the QCL spectroscopic method was employed online to observe simultaneously the spectral signatures of NH_3_OH^+^ and HNO_2_.

## 2. Equipment and Materials

To monitor a chemical process a liquid flow system was established between the reactor vessel and the ATR cell. As the flow passes across the crystal of the ATR cell, the QCL-IR beam irradiates the ATR cell crystal generating an evanescent wave that is in contact with the solution. For this prototype, we used a commercially available Varian HATR base assembly cell with heated flow cell using a Ge 45° coated window from Pike Technologies. Germanium was chosen because it is little affected by HNO_3_. This ATR cell model permits introduction of light from a nonstandard source (i.e., the QCL). The temperature in the ATR work plate was controlled to maintain a constant temperature (±1°C) from the reaction vessel to the ATR. The QCL beam was internally reflected 10 or 20 times, depending on the thickness of the crystal being 4 or 2 mm, respectively.

The QCL system (Daylight Solutions, CA) has four laser modules with wavelength tuning ranges of 3.77-4.49 *μ*m and 6.87-12.50 *μ*m. The QCL sources are broadly tunable and pulsed and provide peak power of up to 400 mW. The QCL module was operated in a pulsed mode of 500 ns, 100 kHz repetition rate, 5% duty cycle, and a scan speed of 0.5 *μ*m/sec. The broadly tunable wavelength range allows the system to cover multiple spectral absorption features or peaks of the chemicals of interest, increasing the sensitivity and selectivity of the method. The outgoing beam from the ATR cell is directed onto an MCT detector from VIGO systems model PCI-3TE. The MCT is a compact three stage thermoelectrically cooled detector optimized to provide high performance in the spectral range from 2 to 13 *μ*m, D*∗* being ≥ 4.5x10^8^ cmHz^0.5^/W at *λ*opt. The detector converts the laser light to an electrical signal with corresponding amplitude per wavelength; this is the QCL power profile used as background. In the presence of chemical species, changes in signal amplitude per wavelength of the QCL give a characteristic absorption spectrum of each analyte and therefore a definitive fingerprint for molecular identification. Using this prototype, chemical identification exhibits as attenuation at specific wavelengths of the QCL-IR beam exiting the ATR cell.

**Figure 1:**
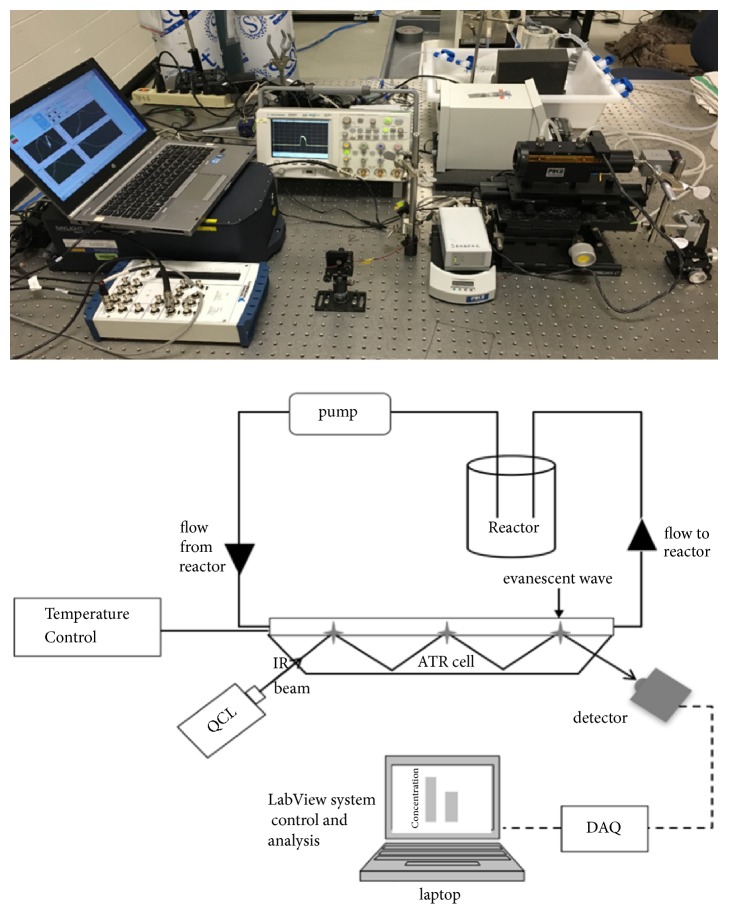
(a) Picture of the ATR-QCL laboratory set up and (b) Schematic of the ATR-QCL apparatus.

A LabVIEW™ [32] interface communicates with the QCL module to control the start/stop of the wavelength scan, data collection, display, and storage. The data is collected using the multifunctional data acquisition NI USB-6251 BNC from National Instruments. The laser module sends a trigger pulse to the DAQ board to begin data capture of each wavelength scan. The start and stop of each scan is determined by the time required for each laser module to tune through its wavelength range. As the QCL emits tuning wavelengths, the LabVIEW interface collects the output voltage from the detector as a function of time. The output voltage of the detector is directly related to the intensity or attenuation of the QCL source per wavelength. The LabVIEW interface uses a Fourier transform to extract the spectral information from the light intensity collected as a function of time at the frequency of the laser pulse rate. The total time from start to end to acquire one spectrum is about 45 seconds with a resolution of 0.6 to 2 nm, about 5 cm^−1^. Thus the spectral resolution of this system is comparable to or better than reported systems for online FTIR spectroscopy [33]. The laboratory setup and schematic diagram are shown in Figures 1(a) and 1(b), respectively.

Solutions of sodium nitrate (10 mM to 2 M), hydroxylamine nitrate (10 *μ*M to 2 M), nitric acid (1-4 M), and sodium nitrite (50 *μ*M to 1 M) were prepared from standard lab reagents (JT Baker batch 45228, Aldrich Lot # 16412DOV, Fisher Lot # 1212072, respectively) without additional purification. Solutions were made up to the mark with deionized water (DI, 18.0 MΩ·cm) in 100 mL volumetric flasks. Solutions were either pipetted directly into the ATR trough or were circulated from a reaction vessel using a peristaltic pump running at 16.5 cm^3^·min^−1^, a flow rate that did not affect the spectra. The reaction vessel, 500 mL, was a custom double-walled glass vessel that could be independently heated using a circulating water bath and was stirred at 200 rpm. Experiments started by preheating reagents to the set temperature before mixing in the reaction vessel. In most cases, the ATR window was also preheated. Reaction times were recorded from the time at which the reagents were mixed in the magnetically stirred reaction vessel. Transit time between the reaction vessel and the ATR was 40s, and so gives the lower bound on time resolution with this configuration. Temperatures ranged from 35 to 80°C, as determined by a NIST standardized SPER Type K thermocouple.

## 3. Results and Discussion

As this is a spectroscopic technique using an ATR liquid cell, a modified Beer-Lambert law can explain the absorption behavior [34]:

(3) $$\begin{array}{c} A=\varepsilon cb^{\prime } \end{array}$$

where* A* is the absorbance, *ε* is the molar absorptivity, and *b*′ is the effective path length. For ATR-FTIR spectroscopy, the effective path length is equal to the number of reflections of the QCL beam in the ATR crystal times the penetration depth.

(4) $$\begin{array}{c} b^{\prime }=Nd_{p} \end{array}$$

**Figure 2:**
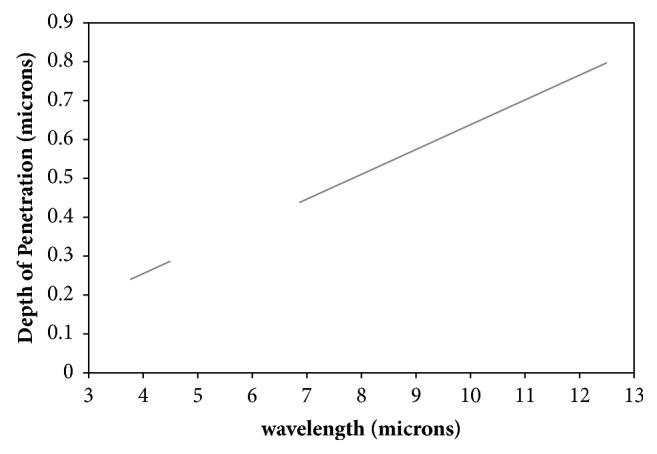
Calculated penetration depth of the QCL wavelength regions, 3.77-12.50 *μ*m for a Ge window.

In (4),* N* is defined as the number of reflections and *d*_*p*_ is the penetration depth at each reflection per wavelength. The penetration depth of the QCL beam is defined as

(5) $$\begin{array}{c} d_{p}=\frac{\lambda_{1}}{2\pi n_{1}{\left[\sin^{2}\theta -{n_{21}}^{2}\right]}^{1/2}} \end{array}$$

where *n*_1_ is the index of refraction of the crystal [35, 36], *n*_2_ is the index of refraction of the sample medium in contact with the crystal [37], *n*_21_ equals *n*_2_/*n*_1_, and *λ*_1_ = *λ*_vaccum_/*n*_1_. Both two and four mm thick germanium windows were used in this experiment.  Figure 2 shows representative penetration depths for a Ge window at room temperature, for our wavelength range of 3.77-12.50 *μ*m.

To analyze the spectral data, five individual wavelength scans of each sample were taken for averaging. Using the average value, the transmission of the sample was corrected for matrix absorption,* I/I*_*o*_, where *I*_*o*_ is the transmittance of water (blank) and* I* is the uncorrected transmittance of the sample. From the corrected transmittance value, the absorption is calculated by

(6) $$\begin{array}{c} A=-\log\frac{I}{I_{0}} \end{array}$$

**Figure 3:**
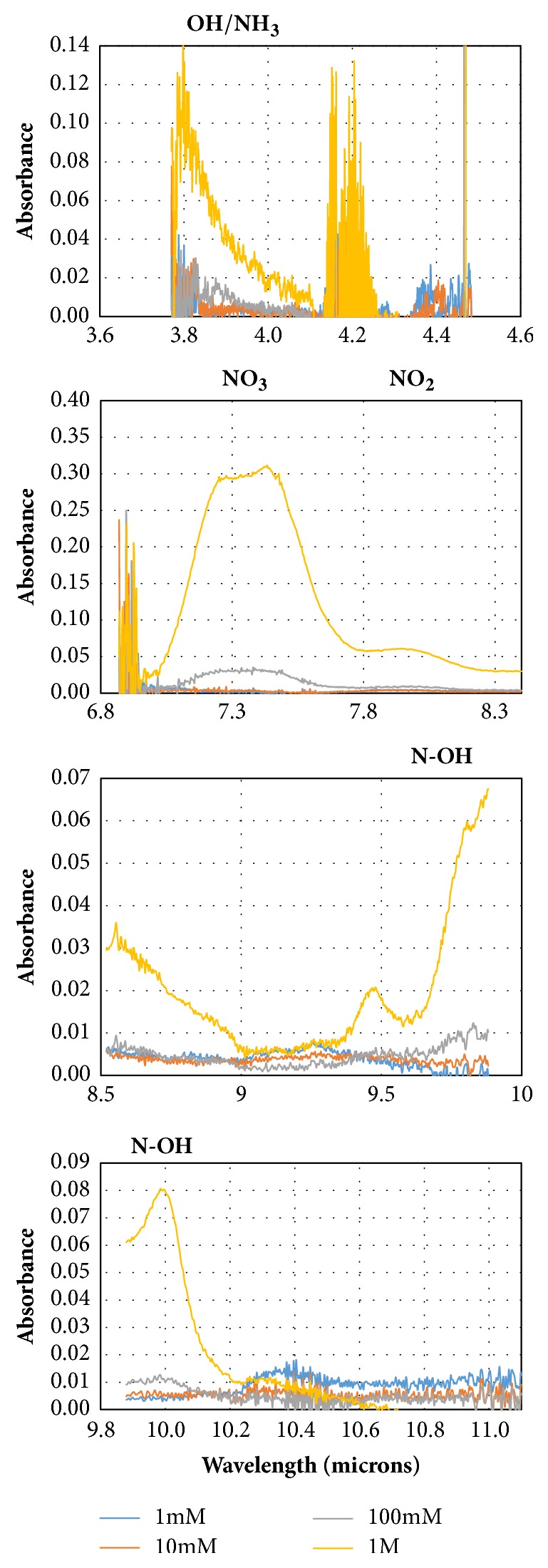
ATR-QCL absorption spectra of hydroxylamine nitrate (1 mM to 1 M) across the spectral range of the four QCL lasers used in this study.

**Figure 4:**
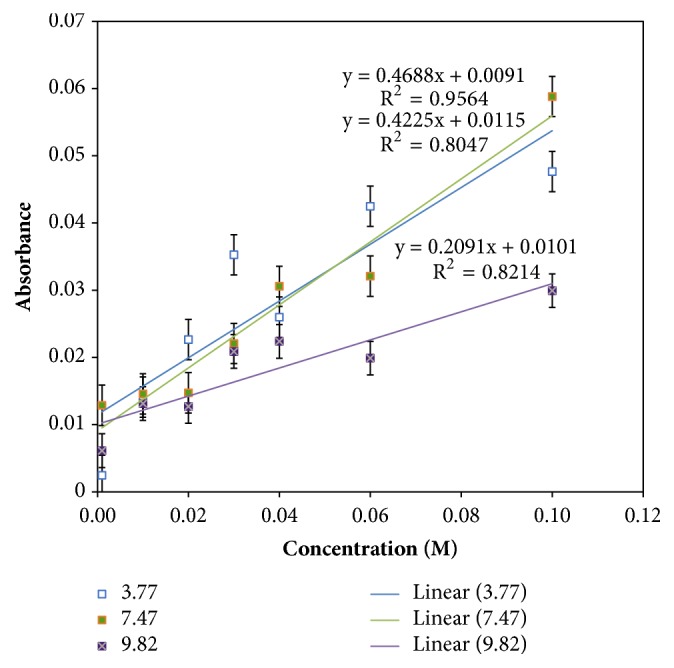
Calibration curves for hydroxylamine nitrate using the QCL-ATR system. The response was very similar for the peaks at 3.77 and 7.46 *μ*m, with the latter giving better linearity. The sensitivity at 9.81 *μ*m was half that seen at the shorter wavelengths.

Absorption spectra of hydroxylamine nitrate (1 mM to 1 M) across the four QCLs are shown in Figure 3. Calibration curves for analytes of interest were prepared using characteristic peak heights or peak areas for various concentrations, as shown in Figure 4. The correlation with concentration was much better for the peak at 7.47 *μ*m than the other absorptions, suggesting that this spectral feature had the least overlap with peaks of other species in solution, although the slopes of the peaks at 7.47 and 3.77 *μ*m are very similar. In this case, with the baseline being pure water, the difference in correlation reflects the signal-to-noise ratio for the peaks at 7.47 and 3.77 *μ*m. The peak at 3.77 *μ*m is at the far blue end of the QCL spectrum where the laser power density is much lower than in the center of the spectrum. The signal-to-noise ratio may also be influenced by the subtraction of the nearby strong absorption for CO_2_. The peak at 9.81 *μ*m appears to have a lower sensitivity to HAN than the other peaks and may show increased noise being located at the limits of the power spectra of two QCLs. It is possible that the choice of a different reference solution, such as HNO_3_ solution, would provide a background spectrum that would give an improved correlation for the peaks at 3.77 and 9.81 *μ*m, but it would not be appropriate for the peak at 7.47 *μ*m.

To calculate the standard deviation on absorption, five intensity ratios from separate scans were used to calculate absorption values using (6). The standard deviation of the noise of the blank at the peak absorption wavelength was then used to calculate the limit of detection (LOD) and the limit of quantification (LOQ) according to IUPAC [38]. The LOD and LOQ are defined as 3.3 *σ*/m and 10 *σ*/m, respectively, where *σ* is the standard deviation and m is the slope of the calibration curve [39]. It has been reported in the literature [40] that the hydroxylamine vibrational spectrum absorbs in the wavelength region covered by our ATR-QCL system, including: the N-OH stretch at 9.7 *μ*m, the NO_3_ combination stretch at 7.4 *μ*m, and the N-H stretch at 3.8-4.1 *μ*m.

Table 1 shows the corresponding calculated values of LOD and LOQ, in grams per liter, for the hydroxylamine nitrate system using the ATR-QCL measurement system.

**Figure 5:**
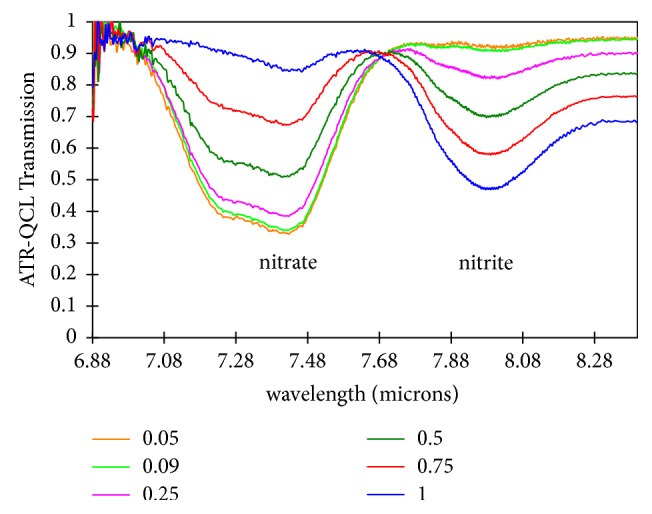
ATR-QCL transmission spectra of nitrite molar fraction range of 0.05-1 in presence of nitrate 1 M solution. The isobestic point at ~7.7 *μ*m is evident from the data.

To demonstrate molecular species detection in high concentration nitrate, we studied the infrared absorption of the aqueous chemical system of NaNO_2_ in the presence of NaNO_3_. Spectra of different concentrations of NaNO_2_ were collected using the QCL-ATR prototype and are shown in Figure 5. The concentration of NaNO_2_ in NaNO_3_ was varied from 0.05 to 1 molar fraction. The absorption peaks correspond well to reference spectra downloaded from the database of the National Institute of Standards and Technology (NIST) [41, 42], although the isobestic point is shifted from 7.60 to 7.65 *μ*m with the QCL system. Regression analysis gave a linear correlation between peak height and concentration, as well as peak area and concentration. Thus, changes in transmission profile can be related to the changes in concentrations in the flow cell. This confirms the capability of the ATR-QCL system to identify multiple species in a chemical system at different concentrations.

To validate the performance QCL-ATR flow system in terms of spectral absorption, an ATR-FTIR spectrum was taken using an FTIR Digilab FTS 7000, equipped with a diamond ATR from Harrick, KBr beam-splitter (Mid-IR), and DTGS detector. An aqueous solution of HAN with concentration of 100 mM was used for this test and the results of both techniques compared. Differences in signal strength were observed for the absorption peaks for the two measurement methodologies. For example, the NO_3_ peak in the FTIR spectra showed a maximum absorbance of 0.007 at 7.0 *μ*m meanwhile the ATR-QCL system showed an absorbance of 0.035 for the same concentration, a factor of five increases in sensitivity. In the case of the NH_3_ peak at 3.7 *μ*m, the FTIR shows an absorbance of 0.002 whereas the ATR- QCL spectroscopy system has an absorbance of about 0.04, a factor of 20 differences in sensitivity.

Sensitivity differences can be attributed to the power of the excitation light source, the optical configuration of the sampling cell, and the choice of detector, i.e., MCT versus DTGS. The sensitivity in the QCL system was also affected by the power spectrum of each laser, giving rise to much lower signal-to-noise at low and high wavelengths. As it was not possible in either case to measure the power entering the cell and that absorbed by the analyte, it is not possible to assign which factor played the most important role. However, the comparison with FTIR-ATR does provide an indication of which factors are important in determining the sensitivity of the QCL-ATR method.

To demonstrate the capability of the system to monitor reactions in real time, the reaction between hydroxylamine nitrate and nitric acid was studied. These species will react quickly at high acid concentrations and at high temperatures [29]. In one configuration, 2 mL of HAN (1M) was placed directly on the ATR work plate preheated to 80°C. Nitric acid was added to the plate via pipette (0.5 mL of 4 M HNO_3_) and the spectrum was monitored over time as the system reacted. However, the use of small volumes and the open plate led to spectral changes that could be mainly attributed to evaporation from the ATR trough.

**Figure 6:**
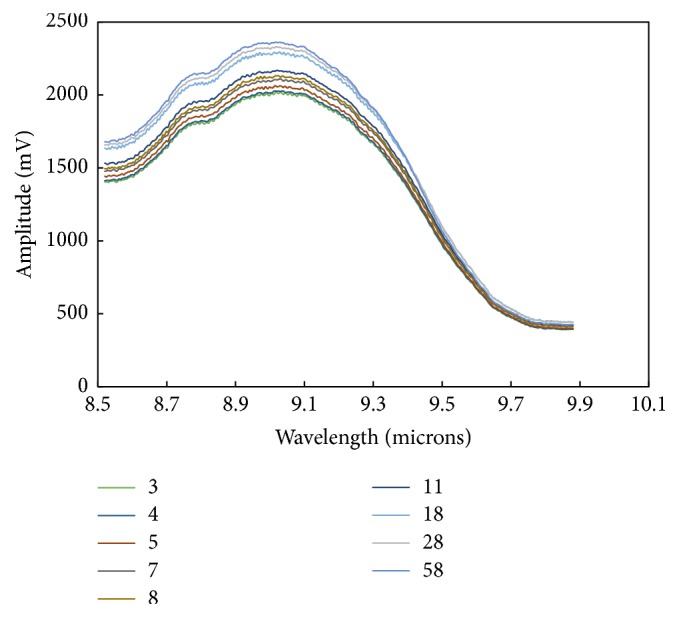
ATR-QCL raw data, signal amplitude (mV) in the absorption region of hydroxylamine nitrate centered at 8.95 *μ*m, taken during the reaction of HAN 0.5M and HNO_3_ 2 M at 80°C. The legend refers to the reaction time in minutes.

The tests were repeated in a flowing system as described earlier, involving 200 mL total volume of reagents, with the nitric acid being added to the hydroxylamine nitrate at the start of the experiment. In this case, a cap on the ATR trough minimized evaporation and the reaction was followed for an hour. The obscuring effects seen with the heated trough plate configuration were not observed. A spectrum of HAN 1 M solution was taken before combining reagents, to enable comparison of absorption behavior before and after addition of HNO_3_. After the HAN spectrum was taken, the reaction was started by the addition of HNO_3_ and the reaction mixture was monitored frequently by taking spectra (up to every 45 s) for an hour. Changes in the spectra observed at specific wavelengths, associated with the N-H and N-OH bonds of the HAN molecule, indicate changes in HAN concentration.  Figure 6 gives raw data from the flowing reaction, in the spectral region from 8.5 to 9.9 *μ*m, or the stretching region in which N-OH bonds absorb.

**Figure 7:**
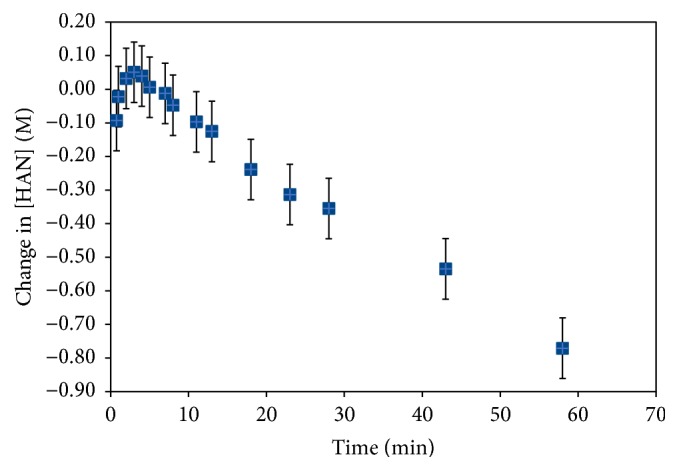
Depletion of HAN as a function of time, from ATR-QCL spectra at 3.77 *μ*m. Final conditions are HAN 0.5M and HNO_3_ 2 M aqueous solution at 80°C.

Figure 7 shows changes in hydroxylamine nitrate concentrations derived from peak heights for the feature at 3.77 *μ*m attributed to the N-H stretch of the amines, directly related to the HAN molecular structure. Final conditions have 0.5 M hydroxylamine nitrate reacting with 2 M HNO_3_ at 80°C. A steady decrease in the concentration of hydroxylamine nitrate was observed from 3 to 60 min, showing consumption of HAN as it reacted with HNO_3_. A lag period and even a slight increase in hydroxylamine concentration were observed, before the expected decrease. The increases could have arisen from effects due to mixing coupled with short-term autocatalysis [23]. These effects may also explain the slightly larger than expected overall decrease in hydroxylamine concentration, but this most likely arose as an artifact from the lag in sampling from the reaction vessel to the ATR cell. The peak at 9.95 *μ*m also showed an abrupt increase in absorption in the first six minutes under the influence of the shoulder of the strong nitrate absorbance from the increase in nitric acid. The peak height then slowly decreased under the influence of hydroxylamine reaction, but the overall effect was much smaller than that of the 3.77 *μ*m feature. Thus, it is useful to be able to compare multiple absorptions, as some may be more appropriate for following a reaction in a particular chemical environment than others. In addition, choice of which background spectrum should be used as *I*_0_ in the equation (6) is critical in determining which spectral effects arise from reagent mixing and sampling and which are representative of the kinetics. Details on the chemical kinetics of hydroxylamine nitrate decomposition will be discussed in a future publication.

## 4. Conclusion

We have developed an approach for high sensitivity and real-time online measurements to monitor chemical processes in aqueous systems. An aqueous reactive system was continuously sampled using mid infrared (Mid-IR) external cavity quantum cascade laser (QCL) high-resolution spectroscopy to detect and identify chemical species in strong nitric acid. The analytical method provides high selectivity, wide dynamic range, and flexibility and can be deployable at a location close to a shielded chemical reactor or “hot cell”.

The sensitivity of the technique to detect small changes in the spectral region due to changes in the chemical molecular structure has been demonstrated. Single or multiple wavelengths can be used to monitor specific absorption behavior of chemical species as a function of time increasing the selectivity of the method. Because infrared spectroscopy provides specific information about the molecular structure, changes observed are specific to each reagent and product and its concentration. This technique can potentially be highly selective to the chemical composition of the aqueous system under investigation, particularly if QCL power spectra are centered on the absorptions of interest. The QCL-ATR configuration was found to provide resolution comparable to an FTIR and provides sufficient sensitivity to detect reactive chemicals, such as hydroxylamine nitrate, in strong (4M) nitric acid.

To perform an analysis of a target nitrate/nitrite system related to the separation of Np and Pu for Pu-238 production, a flow cell was used for continuous sampling of a liquid slipstream. IR spectroscopy was used to detect and identify chemical changes related to the decomposition of HAN in strong nitric acid, an important redox reagent used in the process. Unlike current analytical methods employed in Pu-238 production, this standoff or off-set method does not require the collection of a sample for analysis. Hence, this method can provide online monitoring of the concentration and chemical reactions of HAN, providing information needed for process control.

## Figures and Tables

**Table 1:** Calculated LOD and LOQ (g·L^−1^).

Wavelength (*μ*m)	peak	LOD (g·dm^−3^)	LOQ (g·dm^−3^)
3.778	OH/NH_3_	0.5-1.14	3.45
7.467	NO_3_	0.31-0.634	2.11
9.754	N-OH	1.5-2.94	9.81

LOD=3.3 *σ*/slope, where *σ* = STDEV of the blank.

LOQ=10 *σ*/slope.

## Data Availability

Data are available on request from Marisa Morales-Rodriguez, moralesme@ornl.gov, or from Joanna McFarlane, mcfarlanej@ornl.gov.
